# Synovial-cell sarcoma

**DOI:** 10.11604/pamj.2019.33.291.18741

**Published:** 2019-08-08

**Authors:** Ahmed Nugud, Shomous Nugud

**Affiliations:** 1 Aljalila Children’s Hospital, Dubai, United Arab Emirates; 2 University of Sharjah, Sharjah, United Arab Emirates

**Keywords:** Synovial cell sarcoma, soft tissue tumors, oncology

## Image in medicine

Thirty-nine years old patient presented to the emergency department with left-sided chest pain for four months and 4kg weight loss through the same period. Physical examination showed bulging of the left scapula which is tender to touch and decreased breath sounds on the left side. Chest X-ray done in ED showed opaque left hemithorax with rib resections. A CT was done showing a heterogeneous mass shadow with a solid and cystic component associated with linear calcifications causing a near total collapse of the left lung with shifting of the mediastinum to the right side. Similar findings were seen on an MRI. A PET-CT scan showed hypermetabolic complex mass occupying most of the left hemithorax infiltrating the back muscles bulging to the subcutaneous fat with Irregular thick active gastric mucosa as well and intense large colon activity. A biopsy of the lesion and immunohistochemical studies were done and showed malignant diffuse spindle cell neoplasm positive for CD56, CD99, Bcl2 CKAE1/AE3, H. Caldesmon and EMA. The patient was started on IFOS/DOX chemotherapy regimen and completed three cycles uneventfully.

**Figure 1:**
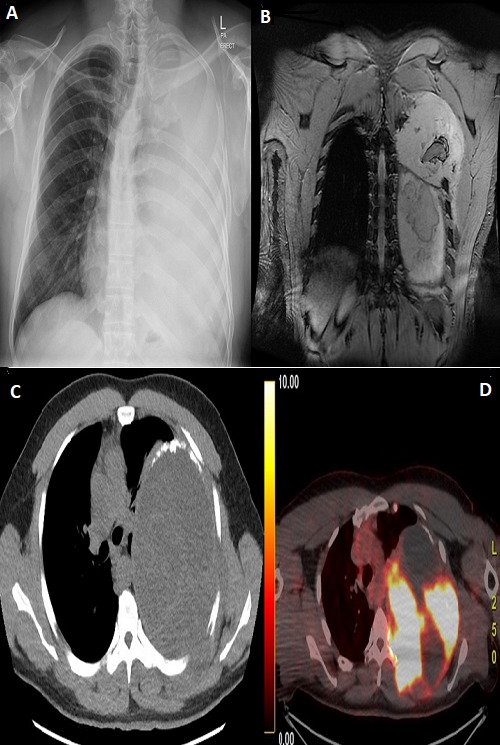
A) left sided chest opacity; B) MRI scan showing total collapse of left lung due to solid homogenous cystic mass invading 3rd, 4th, 5th, 6th ribs with the mass bulging posteriorly infiltrating the left para-spinal musculature; C) CT showing similar findings described on MRI scan in addition to the mass containing solid and cystic parts being inseparable from the chest wall; D) PET CT scan showing hyper metabolic mass involving the left hemithorax

